# Comparison of open and endoscopic carpal tunnel surgery regarding clinical outcomes, complication and return to daily life: A prospective comparative study

**DOI:** 10.12669/pjms.35.6.967

**Published:** 2019

**Authors:** Tahsin Gurpinar, Baris Polat, Ayse Esin Polat, Engin Carkci, Ahmet Sinan Kalyenci, Yusuf Ozturkmen

**Affiliations:** 1 Tahsin Gurpinar, Istanbul Training and Resarch Hospital, Department of Orthopedics and Traumatology, Istanbul, Turkey; 2 Baris Polat University of Kyrenia, Faculty of Medicine, Department of Orthopaedics and Traumatology, Kyrenia, Cyprus; 3 Ayse Esin Polat Dr. Akcicek State Hospital, Department of Orthopaedics and Traumatology, Kyrenia, Cyprus; 4 Engin Carkci, Istanbul Training and Resarch Hospital, Department of Orthopedics and Traumatology, Istanbul, Turkey; 5 Ahmet Sinan Kalyenci, Istanbul Training and Resarch Hospital, Department of Orthopedics and Traumatology, Istanbul, Turkey; 6 Yusuf Ozturkmen, Istanbul Training and Resarch Hospital, Department of Orthopedics and Traumatology, Istanbul, Turkey

**Keywords:** Carpal tunnel syndrome, Carpal tunnel release, Endoscopy

## Abstract

**Objective:**

This study aimed to compare the clinical results and complications as well as patient satisfaction in patients with carpal tunnel syndrome operated with open carpal tunnel release (OCTR) or endoscopic carpal tunnel release (ECTR) techniques.

**Methods:**

This study conducted in Istanbul Training and Research Hospital between August 2016 and January 2018. A total of 54 patients were operated with the ECTR technique and 50 patients were operated with the OCTR technique after failing nonsurgical treatment. Patients functional scores are assessed with the carpal tunnel syndrome-functional status score (CTS-FSS) and carpal tunnel syndrome-symptom severity score (CTS-SSS). Operation time, incision length and complications of the two techniques were noted and compared.

**Results:**

The age, sex distribution, distribution of sides, and complaint period were not significant (p > 0.05) between the groups. The preoperative or postoperative CTS-SSS and CTS-FSS values did not differ significantly (p > 0.05). Incision length, time to return to work and return to daily life in the OCTR group was significantly higher than the ECTR group (p < 0.05).

**Conclusion:**

ECTR has similar results in terms of symptom relief, severity, functional status, pillar pain and complication rates compared to OCTR. However, it has the advantages of early return to daily life, early return to work and less incision length.

## INTRODUCTION

Carpal tunnel syndrome (CTS) is the most common compression neuropathy caused by increased pressure in the carpal tunnel.^1^ It is an important cause of disability and affects both social life and work performance by causing lost days at work and sleep disturbance at night.^2^,^3^ The diagnosis is mainly based on the patient history and physical examination.^4^,^5^ Additional neurophysiological tests can be useful to confirm diagnosis and to give information on the severity of nerve entrapment. When non-operative treatments fail, total release of the transverse carpal ligament (TCL) should be considered to cease the pressure on the nerve.^6^,^7^ In the conventional open carpal tunnel release (OCTR), the surgeon dissects straight down to the flexor tendon retinaculum through a skin incision extending from the wrist creases to the middle of the palm. The flexor retinaculum is then opened and the carpal tunnel is decompressed. On the other hand, Endoscopic carpal tunnel release (ECTR) is mainly performed either through one portal, as described by Agee et al.^8^ or by using two portals, as described by Chow et al.^9^

Less residual pain in the early post-operative period, faster return to work, and fewer wound complications (scar tenderness or hypertrophic scars or infection) are considered to be possible advantages of ECTR; however, increased nerve injury and cost of surgery are major concerns in the literature.^10^,^11^ In this prospective comparative study, we aimed to compare the clinical results and complications as well as patient satisfaction in patients with carpal tunnel syndrome operated with open or endoscopic techniques.

## METHODS

This study conducted in Istanbul Training and Research Hospital between August 2016 and January 2018. A total of 104 patients with carpal tunnel syndrome were prospectively enrolled in this study after failing nonsurgical treatment. Institutional review board approval was granted before initiation of the study, and patients gave informed consent for participation. Patients with the symptoms of numbness or night time pain, sensory impairment or muscle weakness were examined for Phalen’s test and Tinel’s sign. The diagnosis was confirmed if the distal motor latency to abductor pollicis brevis was over 4.5 Ms. Conservative treatment before surgery included anti-inflammatory medication and splinting. The inclusion criteria for surgery are patients who have had CTS complaints for at least 3 months and have not responded to the above-mentioned conservative treatment for at least 3 months. The exclusion criteria were motor deficit, cervical disk pathology, metabolic disease that can cause peripheral neuropathy, history of previous upper extremity surgery in the affected side, a history of a fracture, ligamentous tear, or wrist instability and limitation of movement in the wrist. Patients who agreed to participate in the study were randomly assigned to have open or endoscopic carpal tunnel release. Randomization was performed at initial presentation by assigning odd-numbered medical record identifiers to the open group and even-numbered medical record identifiers to the endoscopic group.

### Surgical procedure

Both of the surgeries were performed under local anesthesia and with tourniquets. At the end of the surgery, a compression dressing was applied from the palm to the wrist. All patients were administered oral analgesics for a week. The sutures were removed at the first follow up two weeks after the surgery.

### Open Technique

**Fig. 1:**
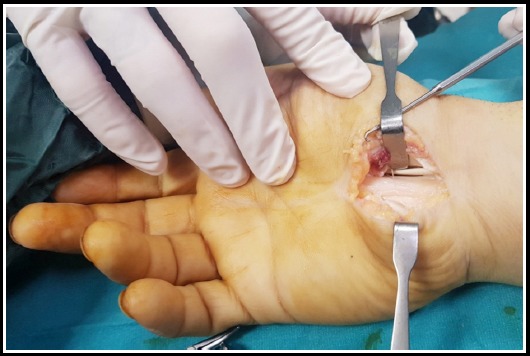
Image of a patient operated by the OCTR technique.

Kaplan cardinal line and the radial border of the fourth ray ending at the wrist crease is marked and a 3-4 cm incision is made over the TCL. A scissor is used to dissect through the subcutaneous fat and palmar tissue. A mosquito clamp is advanced through the carpal canal just deep to the TCL and the TCL is cut from the most ulnar aspect in the canal close to the hook of hamate. After complete release of TCL, the wound is closed (Fig. 1).

**Fig. 2:**
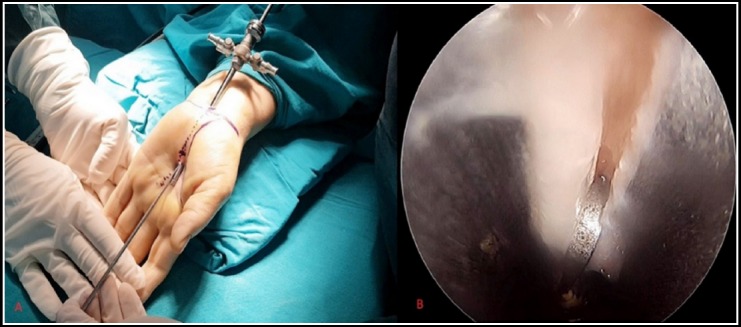
(A): Image of a patient operated by ECTR technique (B): Image of visualization from arthroscope.

The endoscopic procedure was performed through two portals similar to the technique described by Chow et al.^9^ A mini proximal incision is made ulnar to the palmaris longus tendon around 1 cm proximal to the distal wrist crease. The antebrachial fascia is exposed and bluntly spread. The synovial elevator and subsequently the obturator are inserted under the fascia and advanced distally. A stab incision is performed over the obturator’s tip. The obturator is removed and the slotted cannula is left in place. A 30° angle 4-mm rigid scope is inserted through the proximal end of the cannula and advanced distally. The TCL is visualized and a hook knife is advanced from distal to proximally and the TCL is cut from proximal to distally. If necessary, a second or third pass may be performed to transect the ligament completely. The cannula is removed and the wound is closed (Fig. 2).

### Patient Assessment

The patients were evaluated on the day before surgery and at 2 weeks, 6 weeks, 3 months, 1 year and last follow-up time after surgery. Demographic data including age, sex, hand dominance, duration of symptoms, occupation, and previous treatments were questioned and noted preoperatively. In addition, the patients completed a questionnaire that included the Boston questionnaire of the carpal tunnel syndrome-functional status score (CTS-FSS) and carpal tunnel syndrome-symptom severity score (CTS-SSS). Pillar pain was evaluated by application of direct pressure or pinching force on the thenar and hypothenar regions or leaning on a table with the patient’s weight on his/her hands placed on the table’s edge. Complications of the two techniques were noted and compared. The patients were questioned about return to daily life day, return to work day and satisfaction of their operation.

### Statistical Analysis

Mean, standard deviation, median lowest, highest, frequency and ratio values were used in descriptive statistics of the data. The distribution of the variables was measured with the Kolmogorov-Smirnov test. The Mann-Whitney test was used to analyze quantitative independent data. The Chi-square test was used for the analysis of qualitative independent data and the Fisher test was used when the chi-square test conditions were not met. SPSS 22.0 program was used in the analysis.

## RESULTS

**Fig. 3:**
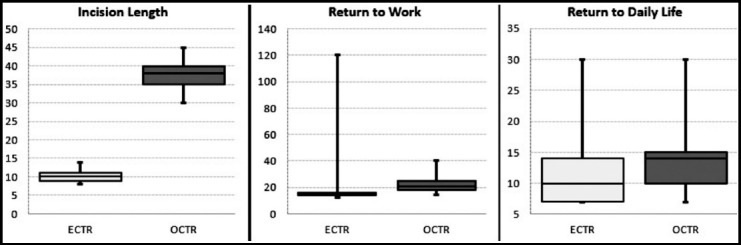
The distribution of statistical different parameters between the two groups.

A total of 54 patients were operated with the ECTR technique and 50 patients were operated with the OCTR technique. The age, sex distribution, distribution of sides, and complaint period were not significant (p > 0.05) between the ECTR and OCTR groups (Table-I). The operation times in the open group and endoscopic group were 18.5 ± 2.5 min and 18.2 ± 2.3 min, respectively. Although the mean operation time in the open group was slightly higher than that in the endoscopic group, there was no significant difference between the two groups (P=0.624). The incision length in the endoscopic group was 10 ± 1.1 mm, which is significantly shorter than that in the open group 37.7 ± 3.3 mm (P = 0.000). In the ECTR and OCTR groups, the preoperative or postoperative CTS-SSS and CTS-FSS values did not differ significantly (p > 0.05). Postoperative CTS-SSS and CTS-FSS values were significantly (p < 0.05) decreased compared to the preop period in both groups. In the OCTR group, the 3rd month, 6th month, and last follow-up pillar pain rates were slightly higher than the ECTR group, but there was no significant difference between the two groups (p > 0.05). In the ECTR and OCTR groups, follow-up time, satisfaction rate or complication rate were not significantly different (p > 0.05). (Table-I). We observed reversible nerve complications in 3 patients in the ECTR group, but no complications related to nerves were seen in the OCTR group. One of these patients suffered from overall exacerbated pain in the hand and numbness on third and fourth fingers for three months and underwent an open revision. In the OCTR group, one patient had a wound infection which did not respond to oral antibiotics and required debridement and IV antibiotics. The time to return to work and return to daily life in the OCTR group was significantly higher than the ECTR group (p < 0.05). (Fig. 3).

**Table I:** Patients demographics, clinical results and complications between ECTR and OCTR groups.

		ECTR				OCTR					

		Mean±sd/n-%			Median	Mean±sd/n-%			Median	p	
Age		51.4	±	7.8	53.0	51.6	±	7.9	52.5	0.873	^m^
Sex	Female	36		66.7%		36		72.0%		0,556	X^2^
	Male	18		33.3%		14		28.0%			
Side	Left	22		40.7%		19		38.0%		0,775	X^2^
	Right	32		59.3%		31		62.0%			
Complaint period (months)		24.0	±	10.9	24.0	24.8	±	10.9	24.0	0.612	^m^
Operation time (minute)		18.2	±	2.3	18.0	18.5	±	2.5	18.0	0.624	^m^
Incision length (mm)		10.0	±	1.1	10.0	37.7	±	3.3	38.0	0.000	^m^
CTS-SSS											
Preoperative		3.2	±	0..4	3..2	3..2	±	0..3	3..2	0..990	^m^
Postoperative		1.4	±	0..3	1..3	1..3	±	0..2	1..3	0..735	^m^
Intra group p		0,000			w	0.000		w			
CTS-FSS											
Preoperative		2,2	±	0.3	2.2	2.2	±	0.3	2.2	0.498	^m^
Postoperative		1,2	±	0.2	1.2	1.1	±	0.1	1.2	0.563	m
Intra group p		0,000			w	0.000		w			
Pillar Pain 3 Months	(-)	50		92.6%		42		84.0%		0.171	X^2^
	(+)	4		7.4%		8			16.0%		
Pillar Pain 6 Months	(-)	52		96.3%		44		88.0%		0.113	X^2^
	(+)	2		3.7%		6			12.0%		
Pillar Pain Last Follow-up	(-)	54		100%		47		94.0%		0.108	X^2^
	(+)	0		0.0%		3			6.0%		
Complication	(-)	51		94.4%		49		98.0%		0.619	X^2^
	(+)	3		5.6%		1			2.0%		
Return to work (day)		17,6	±	14.5	15.0	21.6	±	5.3	21.0	0.000	^m^
Return to Daily Life (day)		10,9	±	4.0	10.0	14.1	±	4.0	14.0	0.000	^m^
Follow-up time (months)		18,0	±	4.5	17.0	18.0	±	4.2	17.0	0.888	m
Satisfaction											
Dissatisfied		2		3.7%		2		4.0%		0.626	X^2^
Rather Satisfied		6		11,1%		8			16,0%		
Very Satisfied		21		38,9%		23			46,0%		
Completely Satisfied		25		46,3%		17			34,0%		

^m^Mann-Whitney u test / ^X²^Chi-square test(Fischer test)

## DISCUSSION

Many studies have investigated the outcomes of open and endoscopic carpal tunnel release.^12^ Sayegh et al.^13^ published a meta-analysis of good-quality randomized controlled clinical trials comparing the two techniques and they concluded that symptom relief, severity, and functional status were similar for both techniques; however, patients who underwent endoscopic surgery had earlier return of grip and pinch strength.

Vasiliadis et al.^14^ reported that open and endoscopic surgery had similar effectiveness in relieving symptoms and improving functional status. In this study, we have compared the clinical outcomes of the endoscopic surgery versus open surgery prospectively. Similar to the literature, the mean symptom severity scores and functional capacity scores of both groups improved significantly; however, they did not differ between groups. On the other hand, we have seen differences between groups in terms of return to work or daily life in favor of endoscopic surgery.

The pillar pain is characterized by pain or tenderness in the thenar or hypothenar eminence or radial and ulnar tenderness, which delays return to work or daily life and causes dissatisfaction. The exact etiology of pillar pain is not clear, but it is reported to be seen between 6% and 36% after carpal tunnel surgery.^15^ We observed pillar pain and scar tenderness in both of our groups, but it was seen considerably more in the OCTR group during the first six months. Polvsen et al.^16^ reported less pillar pain at the end of 3 months following endoscopic release. Similarly, Trumple et al.^17^ reported less scar tenderness during the first 3 months after the endoscopic technique when compared with the open incision technique. In accordance with the literature, in our study, although not statistically significant, less pillar pain was observed in the ECTR group.

One of the promising advantages of ECTR is thought to be early return to work; however, there are controversies in the literature. Scholten previously reported a weighted mean difference in time to return to work as 6 days earlier in the ECTR group than the OCTR group.^7^ Likewise, Chen et al. found that ECTR resulted in 8 days earlier return to work than OCTR.^18^ On the contrary, Thoma analyzed data pooled from three studies and found no significant difference between the two techniques in return to work time.^11^ Our patients returned to work or daily life 9 days earlier in the ECTR group. However, we excluded one of our patients from this calculation since he underwent an open revision surgery and could only return to work 4 months after the first surgery. When we take complications into account, it is hard to state that ECTR reduces the number of off days. The complications may be transient and rare but the consequences may be huge, especially for workers.

Complications including injury to the flexor tendons, median ulnar and digital nerves, and superficial palmar arterial arch have been reported when performing the endoscopic procedure. In this study we observed reversible nerve complications in three patients in the ECTR group; however, no complication related to nerves were seen in the OCTR group. One of these patients suffered from overall exacerbated pain in the hand and numbness on third and fourth fingers for three months and therefore we performed an open revision. In the open surgery, we observed inadequate release of FCL, but there was no neural macroscopic injury. The symptoms of two other patient treated with conservative treatments. A systematic meta-analysis of 13 randomized controlled trials by Thoma et al.^11^ reported that the risk of causing reversible nerve injury with ECTR was three times higher than that with OCTR treatment. However, in a database study, median nerve injury with open CTR was reported to be about 1.9 to 2.9 times more than with endoscopic CTR.^19^ Likewise, Chen et al. reported that the rate of irreversible nerve problems was higher in OCTR hands than ECTR.^18^ They considered that ECTR is safer than OCTR because of the lower rates of irreversible nerve problems.

In the OCTR group, one patient had a wound infection which did not respond to oral antibiotics and required debridement and IV antibiotics. Several studies have found significantly more wound problems (infection, hematoma or wound dehiscence) in patients undergoing open CTR compared to patients undergoing endoscopic CTR.^17^,^20^

### Limitations of the study

There are some limitations to this study. The relatively small sample size and short post-operative follow-up period. We could not compare the effect of these two surgical techniques on hand grip strength. However, we believe that this study is a good example of comparing the clinical results of the two techniques with patient satisfaction, complications, and return to daily life.

## CONCLUSION

In conclusion, ECTR has similar results in terms of symptom relief, severity, functional status, pillar pain and complication rates compared to OCTR. However, it has the advantages of early return to daily life, early return to work and less incision length.

### Authors’ Contribution:

**TG and BP:** Study design, writing and editing manuscript.

**AEP:** Did statistical analysis and interpretation of data.

**EÇ** and **ASK:** Data collection and performed surgery.

**YÖ:** Review and final approval of manuscript.
